# Soluble EpCAM levels in ascites correlate with positive cytology and neutralize catumaxomab activity *in vitro*

**DOI:** 10.1186/s12885-015-1371-1

**Published:** 2015-05-07

**Authors:** Andreas Seeber, Agnieszka Martowicz, Gilbert Spizzo, Thomas Buratti, Peter Obrist, Dominic Fong, Guenther Gastl, Gerold Untergasser

**Affiliations:** 1 Experimental Oncogenomics, Tyrolean Cancer Research Institute, Innsbruck, Austria; 2 Oncotyrol – Center for Personalized Cancer Medicine, Innsbruck, Austria; 3 Department of Hematology and Oncology, Medical University of Innsbruck, Innsbruck, Austria; 4 Division of Vascular Biology, Department of Medical Biochemistry and Biophysics, Karolinska Institute, Stockholm, Sweden; 5 Hemato-Oncological Day Hospital, Hospital of Merano, Merano, Italy; 6 Department of Internal Medicine, Hospital of Merano, Merano, Italy; 7 Pathology Laboratory, Hospital of Zams, Zams, Austria

**Keywords:** Ascites, Catumaxomab, Cytology, Elisa, Liver cirrhosis, Peritoneal carcinomatosis, Soluble EpCAM

## Abstract

**Background:**

EpCAM is highly expressed on membrane of epithelial tumor cells and has been detected as soluble/secreted (sEpCAM) in serum of cancer patients. In this study we established an ELISA for *in vitro* diagnostics to measure sEpCAM concentrations in ascites. Moreover, we evaluated the influence of sEpCAM levels on catumaxomab (antibody) - dependent cellular cytotoxicity (ADCC).

**Methods:**

Ascites specimens from cancer patients with positive (C+, n = 49) and negative (C-, n = 22) cytology and ascites of patients with liver cirrhosis (LC, n = 31) were collected. All cell-free plasma samples were analyzed for sEpCAM levels with a sandwich ELISA system established and validated by a human recombinant EpCAM standard for measurements in ascites as biological matrix. In addition, we evaluated effects of different sEpCAM concentrations on catumaxomab-dependent cell-mediated cytotoxicity (ADCC) with human peripheral blood mononuclear cells (PBMNCs) and human tumor cells.

**Results:**

Our ELISA showed a high specificity for secreted EpCAM as determined by control HEK293FT cell lines stably expressing intracellular (EpICD), extracellular (EpEX) and the full-length protein (EpCAM) as fusion proteins. The lower limit of quantification was 200 pg/mL and the linear quantification range up to 5,000 pg/mL in ascites as biological matrix. Significant levels of sEpCAM were found in 39% of C+, 14% of C- and 13% of LC ascites samples. Higher concentrations of sEpCAM were detectable in C+ (mean: 1,015 pg/mL) than in C- (mean: 449 pg/mL; p = 0.04) or LC (mean: 326 pg/mL; p = 0.01). Soluble EpCAM concentration of 1 ng/mL significantly inhibited ADCC of PBMNCs on EpCAM overexpressing target cells.

**Conclusion:**

Elevated concentrations of sEpCAM can be found in a subgroup of C+ and also in a small group of C- patients. We consider that sEpCAM levels in different tumor entities and individual patients should be evaluated prior to applying anti-EpCAM antibody-based cancer therapies, since sEpCAM neutralizes catumaxomab activity, making therapy less efficient.

**Electronic supplementary material:**

The online version of this article (doi:10.1186/s12885-015-1371-1) contains supplementary material, which is available to authorized users.

## Background

In cancer patients, abnormal accumulation of fluid within the peritoneal cavity results in ascites formation and is frequently accompanied by cancer cell accumulation. Such malignancy-related ascites accounts for 10% of all ascites cases [1]. A disturbed equilibrium between fluid production and drainage due to lymphatic vessel obstruction, hyper-permeability and fluid overproduction is the main reason for accumulation of ascites fluid [2,3]. There are different mechanisms how cancer cells can cause ascites. Some malignancies such as ovarian cancer tend to form peritoneal carcinomatosis [4]. In contrast, colon, gastric, breast and pancreatic cancer patients frequently form ascites due to massive liver metastases with or without peritoneal carcinomatosis [5,6]. In the clinical routine, cytological analysis and biochemical tests are used to confirm peritoneal carcinomatosis or portal hypertension as primary cause for ascites [7]. Malignant ascites caused by peritoneal carcinomatosis is associated with a poor outcome [8,9]. In the last decade, the understanding of the biology of malignant ascites has evolved. Markers, such as Carcinoembryonic Antigen (CEA) or Vascular Endothelial Growth Factor (VEGF) have been described to play a role in the pathogenesis of malignancy-related ascites [10-12]. Frequently, these markers correlate with malignancy and increase the sensitivity of cytology analysis, which is approximately 58 to 75% to predict peritoneal carcinomatosis [13,14].

EpCAM is a tumor-associated membrane marker overexpressed in various epithelial malignancies and it has been reported to enhance tumor signaling and proliferation [15-18]. EpCAM can undergo regulated intra-membranous proteolysis (RIP) thereby, translocating the intracellular domain to the nucleus and shedding the extracellular domain to the extracellular compartment [17]. Consequently, the soluble variant of the extracellular domain of EpCAM (EpEX) has been found in sera of cancer patients [19]. EpEX has been shown to support invasion processes of breast carcinoma cells by supporting c-jun signaling [20]. EpEX has been detected by different ELISA-systems in serum samples of healthy and cancer patients, but no accurate reference levels have been determined so far [21,22]. Due to its broad expression in most frequent epithelial cancers, EpCAM became an attractive antigen for targeted therapies. However, most EpCAM-targeting agents did not hold promise [23] and further studies on predictive biomarkers are necessary to privilege the right patient collectives for EpCAM-targeted therapies [24]. EpCAM gene expression has been observed in cancer cells of approximately 75% of patients with malignant ascites [25] and therefore, the EpCAM-targeting antibody catumaxomab was approved in 2009 by the European Union for the intraperitoneal treatment of patients suffering from malignant ascites. Catumaxomab is a trifunctional antibody binding to EpCAM on epithelial-like tumor cells and CD3+ T cells and can activate with its Fc part monocytes and NK cells. Catumaxomab can lower ascites level and prolong the puncture-free survival of cancer patients and increase their quality of life [26]. Actually, there are no reliable data on the levels of sEpCAM in patients with malignancy-related ascites. Therefore, we analyzed and quantified soluble EpCAM (sEpCAM) in a series of ascites samples. To guarantee specificity and accuracy we developed a sandwich ELISA system based on human recombinant EpCAM spiked into ascites as biological matrix. The ELISA system was validated according to “Guidance for Industry - Bioanalytic Method Validation” for measurement in ascites as biological matrix. Our data gave evidence that sEpCAM can be found in 39% of ascites samples with positive cytology in concentrations significantly higher than in ascites samples with negative cytology or specimens from patients with liver cirrhosis.

To study effects of sEpCAM on catumaxomab activity, we performed antibody-dependent cell-mediated cytotoxicity (ADCC) assays *in vitro*. Our *in vitro* assay confirmed that sEpCAM concentrations found in the C+ cohort of patients are able to neutralize catumaxomab-dependent cell-mediated cytotoxicity.

## Methods

### Patients and specimens

Ascites specimens from 102 patients from the period between 2011 and 2013 were retrieved from the local bio-bank repository. Ascites samples without anticoagulants were centrifuged at 2,000 x g for 10 minutes to separate cellular components from the fluid and cell free supernatants (plasma) were stored at −20°C. This retrospective analysis was approved by the ethic committee of Merano (I) after oral and written informed consents of patients (ethics protocol Nr. 16/2011).

### Generation of lentiviral expression plasmids

The plasmids EpCAM-YFP, EpICD-YFP and YFP in the pEYFP-N1 vector backbone were a generous gift of Dr. Olivier Gires and are described by Maetzel e*t al*. [17] The extracellular part of EpCAM with membrane anchor (EpEX-TM) was cloned by amplifying the fragment by the use of KOD polymerase (Novagen) and specific forward (5-TTA GTG AAC CGT CAG ATC CGC TAG C) and reverse primers (5-GGC GAC CGG TGA AAT AAC CAG CAC AAC). PCR fragments were digested with Nhe-I, Age-I (NEB) and ligated into the predigested p-EYFP-N1 vector (Nhe-I, Age-I, NEB) by the use of the Quick ligation Kit (NEB). Open reading frames were cut out by Nhe-I and Not-I (NEB), polished with Klenow (NEB) and ligated into the pENTR-11 Gateway vector (Invitrogen), predigested with Xmn-I and Eco RV (NEB) and polished with Klenow. All amplified and purified pENTR-11 vectors were sequenced for correct orientation and exclusion of incorporated mutations. pENTR-11 vectors were site-specifically recombined with the pLenti6-V5 DEST vector (Invitrogen) using the Gateway LR Clonase II Pus Enzyme Mix (Invitrogen). The resulting pLenti6 DEST vectors with the EpCAM-YFP, EpEX-YFP, EpICD-YFP and YFP open-reading frames (all amino acid sequences are provided in the Additional file 1: Figure S1) were transformed and propagated in One-Shot Stabl3 bacteria (Invitrogen).

### Generation of lentiviral particles

Lentiviruses were produced in HEK293FT cells by transfecting cells with the pDEST6 vectors and helper plasmid mix (ViraPower, lentiviral support kit, Invitrogen) using Lipofectamine 2000. Lentiviral titers were determined by real time PCR and quantification of woodchuck hepatitis virus posttranscriptional response element expression (WPRE-for: 5-ACTGACAATTCCGTGGTGTT; WPRE-rev: 5-AGATCCGACTCGTCTGAGG).

### Generation of HEK293FT cell lines with different EpCAM domains

Wild type HEK293FT cells were purchased from the ATCC and propagated in DMEM high glucose medium containing 10% bovine calf serum (Hyclone) and 100 IU/mL penicillin, 100 μg/mL streptomycin and 2 mM glutamine (all PAA Laboratories GmbH). HEK293FT cell lines were lentivirally transfected (pDEST6) and stable cell lines selected with 2.5 μg/mL blasticidin (Invitrogen). Transgenic cell lines expressing the reporter YFP after 2 weeks of selection were named HEK ^EpCAM-YFP^, HEK ^EpEX-YFP^, HEK ^EpICD-YFP^ and HEK ^YFP^.

### Generation of recombinant human EpCAM

The EpCAM cDNA (NM_002354, Openbiosystems) was subcloned into the p3x FLAG CMV 14 expression vector (SIGMA Biochemicals) by the use of primers amplifying the extracellular domain of EpCAM (EpCAM-for: 5-TAA GAT ATC CGG CGC GCG CGC AGC; EpCAM-rev: 5-CCG TCT AGA TTT TAG ACC CTG CAT) and the KOD polymerase (Calbiochem). PCR products were purified (PCR-Wizard, Promega) and cloned into the expression vector by the use of Eco-RV, Xba-I and the Quick Ligase Kit (all NEB).

Thereafter, ligated constructs were transformed into chemocompetent Top10 cells (Invitrogen), and propagated for large scale production. Plasmids were purified by the Midi-Prep Kit (Qiagen) and sequenced for correct fusion to the C-terminal FLAG tag.

Plasmids were transfected into HEK293FT (Invitrogen) cells using Lipofectamin 2000 (Invitrogen). For recombinant protein production transfected HEK293FT cells were cultivated in serum-free Ex-cell ACF medium (SIGMA Biochemicals) for 4 days. Supernatants of HEK293FT were prepared by high speed centrifugation (10,000 x g, 20 min, 4°C) and sterile filtered by the use of a 0.2 μm filter (Millipore). Next, recombinant FLAG-fusion protein was purified by affinity chromatography and FLAG-M2 agarose beads (SIGMA Biochemicals). The protein was eluted from column under native conditions by an excess of 3xFLAG peptide (SIGMA Biochemicals). Thereafter, protein was dialyzed against phosphate-buffered saline (Fresenius), purity analyzed by SDS-PAGE with Page Blue Protein staining (Thermo Scientific) and quantified by the QuanitPro TM BCA Assay kit (SIGMA Biochemicals).

### Western/dot blot analysis

Twenty μg of protein extract were denatured, separated by a 4-15% SDS-PAGE (Criterion TGX, Bio-Rad) and transferred to nitrocellulose membrane (Whatman). After blocking the membrane in 5% non-fat milk powder dissolved in PBS with 0.1% Tween, membranes were incubated in 0.5% non-fat milk powder at 4°C overnight with 0.1 μg/mL final concentration of detection antibody (BAF960), 0.1 μg/mL final concentration of capture antibody (MAB9601) or 0.1 μg/mL final concentration of mouse anti-human EpCAM (clone C-10, SCBT). Afterwards, membranes were incubated with a HRP-conjugated rabbit anti-mouse IgG (Dako Cytomation) for capture antibody and a HRP-conjugated rabbit anti-goat IgG (Dako Cytomation) for detection antibody, respectively. Next, a dilution of 1:1,000 for 1 hour at room temperature was prepared. After washing, a chemoluminescent substrate (LumiGLO Reagent and Peroxide, Cell Signaling Technology) was added to the membranes and protein was detected in the Chemidoc XRS station (Biorad Laboratories).

For Dot Blot analysis 10 ng of FLAG-tagged EpCAM produced in HEK293FT cells dissolved in assay buffer (1%BSA/PBS) or serum (n = 6, healthy probands) or ascites (n = 12) were spotted onto nitrocellulose membrane.

### Real-time confocal microscopy

Confocal Microscopy was performed with a spinning disk confocal system (UltraVIEW VoX; Perkin Elmer) connected to a Zeiss AxioObserver Z1 microscope (Zeiss). Images were acquired with the Velocity software (Perkin Elmer) using a 63x oil immersion objective with a numerical aperture of 1.42. Images shown are z-stacks of 5 planes with a spacing of 1 μm.

### EpCAM enzyme-linked immunosorbent assay

96-well plates were coated with mouse monoclonal antibody (R&D systems MAB9601) overnight at room temperature in PBS pH 7.2 - 7.4. After wash steps with 0.05% Tween® 20 in PBS the plate was blocked with an assay buffer (1% BSA in PBS, pH 7.2 – 7.4) for 1 hour at room temperature. Recombinant human EpCAM standard was used in a concentration range between 75 – 5,000 pg/mL and ascites samples were measured in a dilution of 1:10 in an assay puffer and incubated for 2 hours at room temperature. After repeated washing steps a biotin-labelled goat-detection antibody (R&D systems, BAF960) was added to each well and incubated for another 2 hours at room temperature. Thereafter, streptavidin-HRP was added and incubated in the dark for 20 minutes at room temperature. After final washing steps, a substrate solution (1:1 mixture of H_2_O_2_ and tetramethylbenzidine) was added to each well and incubated in the dark for another 20 minutes at room temperature. As stop solution 2N H_2_SO_4_ was used. Color development was detected at a wavelength of 450 nm and 570 nm using microplate reader (TECAN, Infinite F50). A seven point standard curve was used to calculate the amount of EpCAM (pg/mL) in ascites samples.

### Antibody-Dependent Cell-mediated Cytotoxicity (ADCC) and flow cytometry

Catumaxomab-dependent cell-mediated cytotoxicity assay were performed with peripheral blood mononuclear cells (PBMNCs) and EpCAM-YFP or EpICD-YFP transfected HEK293FT cell lines. PBMNCs were collected and isolated from healthy donors by Lymphoprep™ (LSM-separating solution, PAA) according to the manufacturer’s instruction. EpCAM-YFP and EpICD-YFP HEK293FT cells were propagated in 6 well (5 × 10^5^ cells/well). One day after, medium was changed and replaced with fresh culture medium, containing 10% serum and 1 ng/mL catumaxomab (Neovii, Fresenius Biotech) and increasing concentrations of sEpCAM (0.2 - 5 ng/mL). To obtain a 1:10 ratio between target and effector cells 5 × 10^6^ PBMNCs were added into each well and cell mixtures incubated for 24 h at 37°C. ADCC reaction was documented by a Fluorescence Microscope (Zeiss, Axiovert 200) by the use of the Axiovision Software. Thereafter, all cells were detached by accutase solution (Sigma Biochemicals), washed, resuspended in PBS and measured on the flow cytometer (BD FACSCanto™ II, BD Biosciences). YFP- positive HEK cells were detected at a wavelength of 488 nm, counted and analyzed by the BD FACS DIVA software.

Human diploid fibroblasts (HDF) were purchased from Promocell and HRT-18 cells from ATCC. All used cells lines were authenticated by us (STR-profiling). All cell lines were cultivated in RPMI 1640 medium (Sigma Biochemicals) with 10% bovine calf serum (Hyclone) and 100 IU/mL penicillin, 100 μg/mL streptomycin and 2 mM glutamine (all PAA Laboratories GmbH). HDFs and HRT18 cells were seeded at a density of 50,000 cells/cm^2^ and were propagated overnight for ADCC assays. Catumaxomab (1 ng/mL) was pre-incubated for 30 min with a pool of EpCAM high (5,000 pg/mL) and EpCAM low ascites (< 200 pg/mL) diluted 1:4 in culture medium, thereafter added to tumor cells. After 1h of incubation freshly isolated PBMNCs were added in a 1:10 target to effector cell ratio. Cells were detached after 24 h and collected for staining with Fluorescein-labeled mouse anti-human EpCAM (R&D Systems) and Annexin V apoptosis detection kit PerCp-eFluor 710 (E-Biosciences). After 20 min of staining, cells were washed and measured on the flow cytometer (BD FACSCanto™ II, BD Biosciences). Percentages of apoptotic cells were determined after gating for EpCAM ^+^/Annexin V ^+^ cells.

### Statistical analysis

For statistical analysis of data the Statistical Package for the Social Sciences (SPSS, Chicago, IL, USA) version 11.5 and Graph Pad Prism 5 (Graph Pad Software Inc.) were used. Chi-square, student t-test and Mann–Whitney-test were used. P-values below 0.05 were defined as statistically significant.

## Results

### Development of the EpCAM ELISA system with recombinant EpCAM

**Figure 1:**
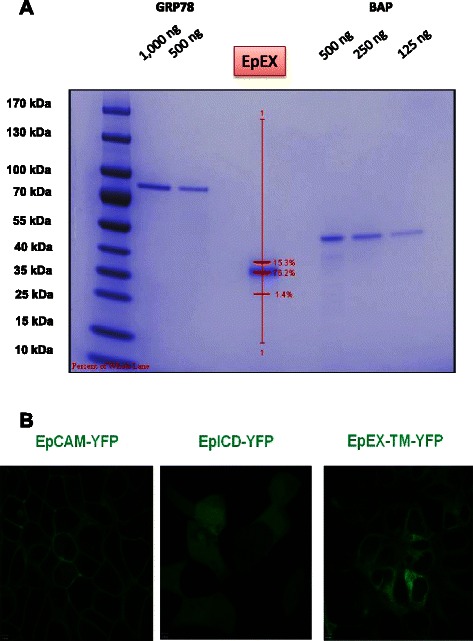
EpCAM standard and stable control cells lines for ELISA. **(A)** Coomassie brilliant Blue staining of PAGE gel in the presence of two different standard proteins (GRP78 and BAP). Purity of the EpEX was approx. 93% as determined by Page Blue staining and densitrometric analysis. Molecular weight marker indicates reference proteins in KDa. EpEX-FLAG (293 aa) has an estimated size of 35 KDa. **(B)** Real-Time confocal microscopy pictures of living HEK293FT cell lines expressing different EpCAM isoforms fused to YFP: EpCAM-YFP shows expression only on cell membrane, EpEX-TM-YFP is detectable in the endoplasmatic reticulum and on cell membrane. Due to the lack of transmembrane region EpICD-YFP is found in cytosol and nucleus of living cells.

We produced recombinant human EpCAM protein in human HEK293FT cell line to ensure correct folding and glycosylation patterns similar to that found in human tumor cells (protein sequence can be found in Additional file 1: Figure S1). Recombinant EpCAM showed a purity of approximately 93% as shown by Page blue staining and densitrometric analyses (Figure 1A).

 To analyse specificities of the antibodies used, we generated HEK293FT cell line stably expressing different domains of EpCAM in fusion with yellow fluorescent protein (YFP) and named them HEK ^EpCAM-YFP^, HEK ^EpEX-YFP^, HEK ^EpICD-YFP^ and HEK ^YFP^. This enables us to localize all protein isoforms in living cells by confocal microscopy (Figure 1B).

**Figure 2:**
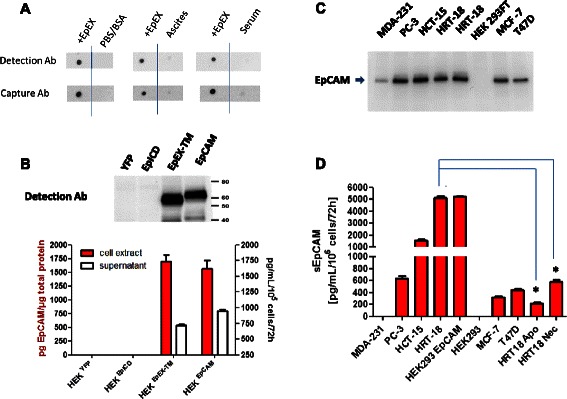
Specificity of EpCAM ElISA. **(A)** Dot Blot analysis of used detection antibodies to react with spiked native recombinant EpEX standard (10 ng) in assay buffer (PBS/1%BSA), serum (pool of 6 healthy donors) and ascites (pool of 12 negative patients). Capture and detection antibodies specifically react with EpEX in all biological matrices used and gave low background in serum and ascites **(B)** Detection antibodies bind specifically to EpCAM and EpEX-TM in established HEK-293FT cell lines expressing different variants of EpCAM or the control protein YFP (Western Blot). Results were confirmed by sandwich ELISA in cell extracts and respective supernatants. Concentrations are calculated for 1 μg total cellular protein extract. sEpCAM levels in supernatants of the different HEK cell lines were calculated with regard to cell number, culture volume and time. **(C)** Western Blot analysis of EpCAM expression in different human cancer cell lines under standard culture conditions **(D)** Concentrations of sEpCAM in supernatants were calculated with regard to cell number, culture volume and time. HRT-18 ^Apo^ and HRT-18 ^Nec^ indicate supernatants of HRT-18 cells that underwent apoptosis (100 μM A23187) or necrosis (100 mM H_2_O_2_).

For the detection of sEpCAM we used two antibodies to establish a sandwich ELISA system. To analyze their specificities for native 3D-EpCAM, we spiked our recombinant EpCAM protein in EpCAM negative ascites and serum samples as biological matrices and tested them in Dot Blot analysis (Figure 2A). Both capture and the detection antibodies are recognizing recombinant human EpCAM protein (10 ng) in EpCAM free assay puffer, ascites and serum specimens. Furthermore, specificities of detection antibodies were tested under reducing conditions by Western Blot analysis (Figure 2B). Detection antibodies are recognizing specifically HEK ^EpCAM-YFP^ and HEK ^EpEX-YFP^, but not HEK ^EpICD-YFP^ or HEK ^YFP^. High amounts of EpCAM were monitored only in HEK ^EpCAM-YFP^ and HEK ^EpEX-YFP^ cytosolic extracts and the respective supernatants (Figure 2B). Moreover, we were able to detect sEpCAM in supernatants of human epithelial-like cancer cell lines that expressed EpCAM in Western Blot analysis (Figure 2C/D). Levels were from undetectable (<200 pg/mL) in breast carcinoma line MDA-231 up to 5,000 pg/mL in the colorectal carcinoma line HRT-18. Interestingly, when HRT-18 cells underwent apoptosis or necrosis sEpCAM levels significantly decreased (Figure 2D).

### Validation of the EpCAM ELISA system

**Figure 3:**
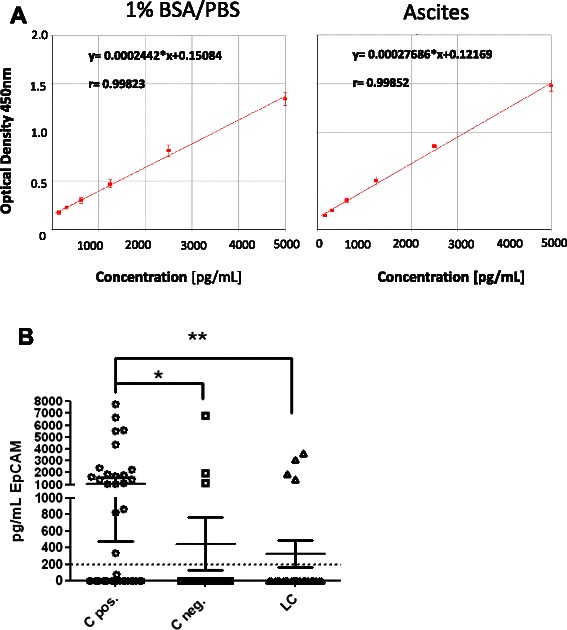
Measurement of sEPCAM in ascites. **(A)** Linear functions with recombinant EpCAM standard (156 pg - 5,000 pg/mL) spiked in ascites as biological matrix. **(B)** Scatter dot blot analysis with 95% confidence interval of the three ascites etiologies (C pos., C neg., LC). Soluble EpCAM concentrations were significantly different between C pos. and C neg. (*p < 0.05) and between C pos. and LC (**p < 0.05).

Recombinant human EpEX protein produced in HEK293FT cells was used as standard in this EpCAM ELISA system. A standard calibration curve was drawn to permit quantification of soluble EpCAM in ascites (Figure 3A). Regarding recovery rates of spiked recombinant EpCAM we got a mean recovery rate of 93% in ascites samples (Additional file 2: Material 1). We achieved a linear quantification range of 200 to 5,000 pg/mL for ascites samples (Figure 3A).

To validate the accuracy of our results, we analysed intra- and interassay coefficient of variation (CV). We obtained an intra- and interassay CV of 3.2% and 4.5%, respectively (Additional file 2: Material 2 and 3) .Moreover, we evaluated short-, long-term and the freeze-and-thaw stabilities of ascites samples (Additional file 2: Material 4). Repeated freezing-and-thawing cycles resulted in a mean sample degradation of 5.3% (Additional file 2: Material 5). Evaluation of long term- stability at −20°C resulted in a mean degradation of 11.2% (Additional file 2: Material 6).

### Soluble EpCAM concentrations in ascites samples

In this study we collected 102 ascites samples deriving from patients suffering from cancer (n = 71) or liver cirrhosis (n = 31). Hence, we obtained 49 patients (69.0%) with positive cytology (C+) and 22 (31.0%) patients with negative cytology (C-). Table 1 shows the cancer entities that produced ascites with positive and negative cytology.

**Table 1:** **Cancer entities positive or negative for tumor cells in ascites; CUP = Carcinoma of Unknown Primary; HCC = Hepatocellular Carcinoma**

Cytology		
	Positive	Negative
Tumor entity	% (n)	% (n)
Ovarian	28.7 (14)	9.1 (2)
Pancreas	14.3 (7)	9.1 (2)
Stomach	14.3 (7)	4.5 (1)
Breast	12.2 (6)	13.6 (3)
Colorectal	6.1 (3)	22.8 (5)
Lung	6.1 (3)	9.1 (2)
CUP	6.1 (3)	-
HCC	-	18.2 (4)
Others	12.2 (6)	13.6 (3)
**Total**	**100 (49)**	**100 (22)**

We analyzed all 102 cell-free ascites samples with our validated EpCAM ELISA system. Twenty-six (25.5%) samples were found to be sEpCAM positive (Table 2).

**Table 2:** **Soluble EpCAM levels compared with ascites etiologies**

		Soluble EpCAM level				
	Total	> 200 pg/mL		< 200 pg/mL		
	n	n	%	n	%	*P*
**Whole Samples**	102	26	25.5	76	74.5	
**Etiology**						
C Positive	49	19	38.8	30	61.2	0.034
C Negative	22	3	13.6	19	86.4	0.013
Liver Cirrhosis	31	4	12.9	27	87.1	0.938
**Entity**						
Ovarian Cancer	16	7	43.8	9	56.3	
Non-Ovarian	55	15	27.3	40	72.7	0.210
Cancer						

We compared than sEpCAM status between the three ascites cohorts (Table 3, Figure 3B). Soluble EpCAM expression correlated significantly with positive cytology (p = 0.034). In the C+ cohort sEpCAM was positive in 38.8% (n = 19). In contrast, sEpCAM showed a positivity of only 13.6% (n = 3) in the C- cohort. The difference was even higher when we compared C+ with ascites from liver cirrhosis (p = 0.013). Moreover, we observed a strong correlation of positivity in ascites and the respective serum samples (Table 4).

**Table 3:** **Soluble EpCAM concentrations in ascites specimens: Mean and range**

Ascites origin	Mean [pg/mL]	Range [pg/mL]
**Cytology positive (C+)**	1,015	LLOQ – 7,750
**Cytology negative (C-)**	449	LLOQ – 6,819
**Liver Cirrhosis (LC)**	326	LLOQ – 3,655

**Table 4:** Soluble EpCAM concentrations in ascites and respective serum samples of patients (n = 10)

Patient [Entity]	Ascites [pg/mL]	Serum [pg/mL]
**Patient 1 [Pancreatic]**	< LLOQ	< LLOQ
**Patient 2 [HCC]**	< LLOQ	< LLOQ
**Patient 3 [NET]**	< LLOQ	< LLOQ
**Patient 4 [Ovarian]**	3,477	1,977
**Patient 5 [Ovarian]**	< LLOQ	< LLOQ
**Patient 6 [Pancreatic]**	< LLOQ	< LLOQ
**Patient 7 [Gastric]**	2,440	765
**Patient 8 [Ovarian]**	5,575	2,348
**Patient 9 [Gastric]**	< LLOQ	< LLOQ
**Patient 10 [Ovarian]**	3,650	7,750

### Analysis of neutralizing effect of sEpCAM on catumaxomab

**Figure 4:**
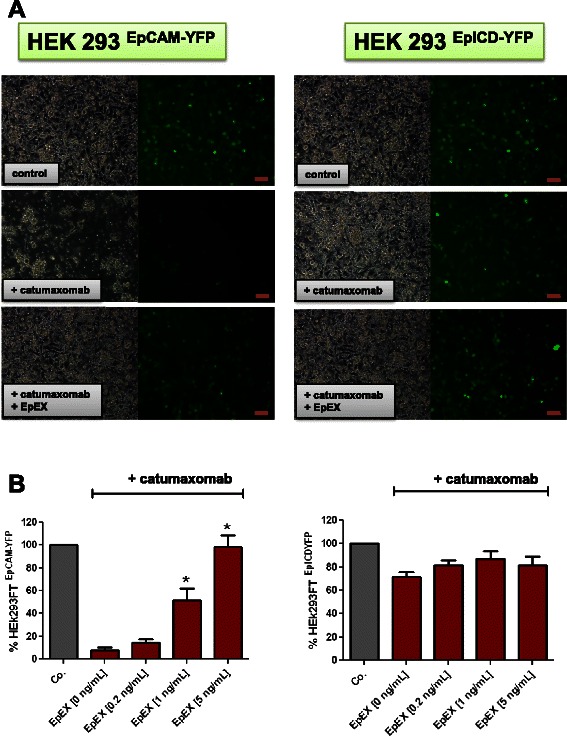
Analysis of ADCC in control cell lines. Effects of sEpCAM on catumaxomab-dependent cell mediated cytotoxicity **(A)** HEK ^EpCAM-YFP^, and HEK ^EpICD-YFP^ cells were incubated without or with 1 ng/mL catumaxomab together with a 10-fold excess of human PBMNCs. PBMNCs with catumaxomab were efficiently killing EpCAM-YFP cells, but did not attack EpICD-YFP cells. Living cells are displayed by fluorescence microscopy (left picture). Incubation with 1 ng/mL EpEX already inhibited catumaxomab -dependent cell mediated cytotoxicity on EpCAM-YFP overexpressing HEK cells. Bars indicate 100 μm. **(B)** HEK ^EpCAM-YFP^, and HEK ^EpICD-YFP^ cells were incubated without or with 1 ng/mL catumaxomab together with a 10-fold excess of human PBMNCs and increasing concentrations of EpEX (0, 0.2, 1, 5 ng/mL). YFP positive events were counted by flow cytometry (488 nm) and normalized to control cells without catumaxomab/PBMNC incubation (100%). ADCC was repeated three times (Mean ± SEM).

We hypothesized that sEpCAM in ascites could interfere with the anti-EpCAM antibody catumaxomab used for treatment of patients. To investigate these interactions we conducted a catumaxomab-dependent cell mediated cytotoxicity assay (ADCC) *in vitro* with concentrations of antibody used in patients (1 ng/mL) and tested increasing concentrations of sEpCAM (up to 5 ng/mL as observed in a collective of C+ patients). HEK ^EpCAM-YFP^ and HEK ^EpICD-YFP^ were used as target cells and peripheral blood mononuclear cells (PBMNCs) as effector cells. Soluble EpCAM at concentrations of 1 ng/mL was neutralizing catumaxomab-dependent cell mediated cytotoxicity in HEK ^EpCAM-YFP^ cells (Figure 4A). HEK ^EpICD-YFP^ cells served as negative control and these cells were not lysed by catumaxomab-dependent cell- mediated cytotoxicity, showing that catumaxomab is binding specifically to EpEX and not other surface molecules (Figure 4A). A more detailed analysis of ADCC by counting YFP+ cells by flow cytometry revealed that approx. 90% of HEK ^EpCAM-YFP^ cells were lysed without sEpCAM. (Figure 4B, left image). HEK ^EpICD-YFP^ control cells were protected from ADCC even without EpEX (Figure 4B, right image). In the presence of 1 ng/mL or 5 ng/mL EpEX protein nearly 50% or 95% of cells were protected from ADCC (Figure 4B, left image).

**Figure 5:**
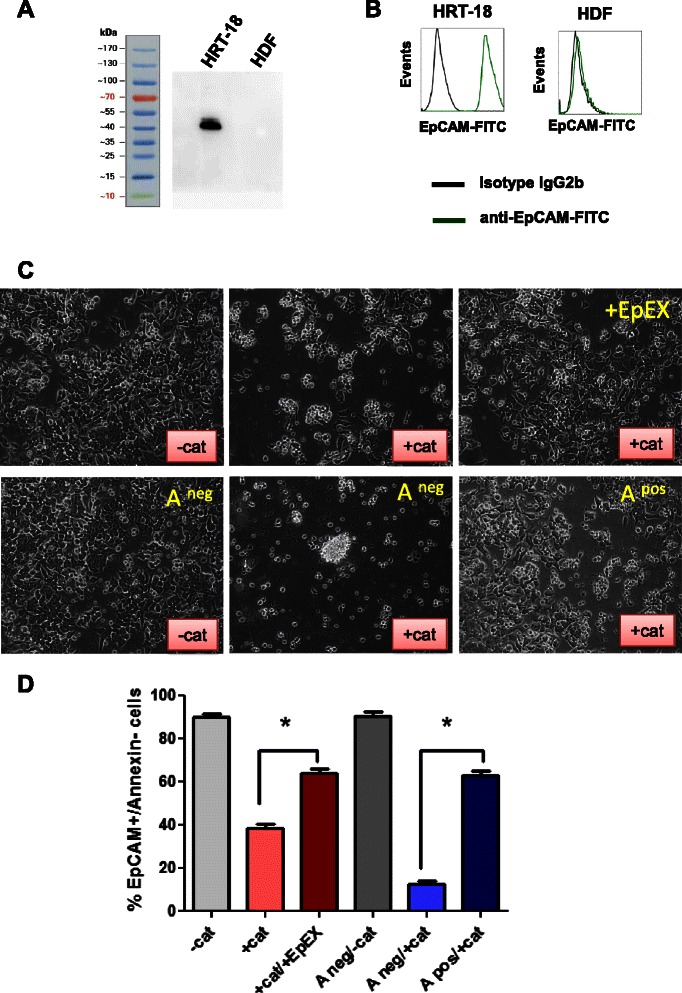
Soluble EpCAM in ascites inhibits ADCC of tumor cells. Effects of sEpCAM/EpEX on catumaxomab-dependent cell mediated cytotoxicity **(A)** Colorectal carcinoma cells HRT-18 and human diploid fibroblasts (HDFs) were analyzed by Western Blot for the expression of EpCAM. **(B)** Flow cytometry analysis of EpCAM expression on HRT-18 cells and HDFs. **(C)** HRT-18 cells were incubated with or without 1 ng/mL catumaxomab (cat.) together with a 10-fold excess of human PBMNCs (lymphocytes and monocytes). PBMNCs with catumaxomab were efficiently killing cells, but did not attack HRT18 cells in the presence of 5 ng/mL recombinant EpEX (upper panel). Experiments were repeated with catumaxomab and sEpCAM positive (A ^pos^, 5 ng/mL) and negative (A ^neg^ <200 pg/mL) ascites pools (lower panel). ADCC was inhibited in ascites samples with high sEpCAM levels. **(D)** Viable tumor cells are displayed as EpCAM^+^/Annexin^−^ cells after staining and analysis by flow cytometry. EpEX and ascites with high concentrations of sEpCAM inhibited catumaxomab-dependent cell mediated cytotoxicity (Mean ± SEM).

ADCC experiments were repeated with EpCAM ^high^ HRT-18 and human diploid fibroblasts (HDFs) having no detectable EpCAM expression on Western Blot or flow cytometry analysis (Figure 5A/B). HDFs were protected from catumaxomab-mediated ADCC (data not shown). Catumaxomab mediated ADCC in HRT-18 cells was significantly inhibited by 5 ng/mL recombinant EpEX in standard culture medium (Figure 5C, upper panel). The same significant inhibition could also be observed with ascites having 5 ng/mL sEpCAM, whereas catumaxomab-mediated ADCC of tumor cells was efficient in sEpCAM negative ascites (Figure 5C, lower panel). Flow cytometry analysis of viable HRT-18 cells (EpCAM ^positive^/Annexin ^negative^ ) revealed that the fraction of viable tumor cells significantly increased in ascites samples with high concentrations of sEpCAM and after addition of recombinant EpEX (Figure 5D).

## Discussion

The detection of the soluble EpCAM protein in body fluids might be used for the estimation of EpCAM ^high^ tumor cells in malignant ascites. Catumaxomab, a bispecific (anti-EpCAM x anti-CD3) trifunctional monoclonal antibody, was approved by the EMA for the intraperitoneal treatment of malignant ascites. However, the selection of patients for this treatment is challenging. The life expectancy is often very short and there is a need for verification of additional parameters, like EpCAM expressing tumor cells that may help to identify patients who benefit most from catumaxomab treatment. Cytology and biochemical analysis are indispensable exams to distinguish peritoneal carcinomatosis from portal hypertension [3,27]. Nevertheless, the sensitivity of cytology testing is estimated to detect only 58-75% of the real cases of peritoneal carcinomatosis cases [13,14]. Furthermore, due to the low sensitivity of cytology it could be possible that our sEpCAM positive samples of patients with negative cytology (n = 4), are in reality patients suffering of peritoneal carcinomatosis having no detectable amounts of tumor cells (false-negative cases). In line with this hypothesis, we observed a broad difference in samples with positive cytology compared to those with a negative cytology. (p = 0.04). Thus, we believe that sEpCAM levels reflect the EpCAM ^high^ tumor cell load in ascites, but our sEpCAM ELISA fails to detect EpCAM ^low^ tumor cells. Noteworthy, higher sEpCAM levels were found only in 39% of patients with tumor cells in ascites. This is by far lower than EpCAM gene expression rates described for tumor cells of malignant effusions [25] and for cell surface protein studies using microfluidic chips [28]. This may implicate that many tumor cells in ascites have probably lost EpCAM expression because they underwent epithelial to mesenchymal transition (EMT, such as MDA231 cells) and tumor cell types releasing only low amounts of sEpCAM into their microenvironment. Noteworthy, we found sEpCAM also in some patients with liver cirrhosis and in these cases (n = 4) may be a surrogate marker for liver regeneration [29,30]. Interestingly, the Oncomine data base search revealed a 28.7 fold-higher EpCAM gene expression in cirrhotic than normal liver tissue (data not shown).

Actually, we have evidence that soluble EpCAM can exist in two different forms: first as variant cleaved on the membrane of EpCAM positive tumor cells (EpEX), second, as full-length protein (EpCAM) expressed on tumor-derived exosomes [31]. Our measurements were done without removal of exosomes from plasma. Therefore, our assay is not able to distinguish between soluble EpCAM and full-length EpCAM found in membranes of exosome vesicles.

It should be critically mentioned, that our population was very heterogeneous and retrospective. More patients with ovarian cancer were in the cytology positive cohort, whereas in the cytology negative group more patients with colorectal cancer were analyzed. So the interpretation of these data should be used with caution and further prospective studies will focus on different tumor entities. In addition, no data about therapies and survival were obtained from these patients. In fact, our scope was to test the diagnostic value of sEpCAM in ascites for peritoneal carcinomatosis. Based on our findings, future studies are on the way to evaluate the prognostic and predictive value of high sEpCAM levels in cancer patients, especially in catumaxomab-treated patients.

In fact, our *in vitro* experiments demonstrated that sEpCAM is neutralizing the activity of catumaxomab in concentrations that we found in a cohort of our patients. Thus, soluble EpCAM is binding to the EpCAM-binding Fab domain of catumaxomab and is blocking this part of the antibody for interaction with the cell membrane of the tumor cell.

## Conclusions

Elevated concentrations of sEpCAM can be found in a subgroup of C+ and also in a small group of C- patients. We consider that sEpCAM levels in different tumor entities and individual patients should be evaluated prior to applying anti-EpCAM antibody-based cancer therapies, since sEpCAM neutralizes catumaxomab activity, making therapy less efficient.

## Additional files

Additional file 1: Figure S1. Amino acid sequences of EpCAM variants.
Additional file 2: Material S1-S6. Validation of the EpCAm ELISA.

## Abbreviations

ADCC: Antibody-dependent cell-mediated cytotoxicity
BAP: Bacterial alkaline phosphatase
CD3: Cluster of differentiation 3
CEA: Carcinoembryonic antigen
CUP: Carcinoma unknown primary
CV: Coefficient of variation
EpCAM: Epithelial cell adhesion molecule
EpEX: EpCAM, Extracellular domain
EpICD: EpCAM, Intracellular domain
GRP78: Glucose-regulated protein 78
HCT-15: EpCAM ^High^ colorectal carcinoma cell line
HEK293FT: Human embryonic kidney 293FT
HRT-18: EpCAM ^High^ colorectal carcinoma cell line
HCC: Hepatocellular cancer
LC: Liver cirrhosis
MDA-231: EpCAM ^Low^ breast carcinoma cell line
MCF-7: EpCAM ^High^ breast carcinoma cell line
PBMNCs: Peripheral blood mononuclear cells
PC-3: EpCAM ^High^ prostate carcinoma cell line
PS-2: Presenelin-2
PAGE: Polyacyl amid gel electrophoresis
C+: Cytology positive
C-: Cytology negative
PCR: Polymerase-chain reaction
sEpCAM: Soluble EpCAM
SDS: Sodium dodeycl sulfate
TACE: Tumor necrosis factor converting enzyme
T47D: EpCAM ^High^ breast carcinoma cell line
TM: Transmembrane
VEGF: Vascular endothelial growth factor
YFP: Yellow fluorescent protein
