# Supplementary material for: Soluble EpCAM levels in ascites correlate with positive cytology and neutralize catumaxomab activity in vitro
Source: BMC Cancer. 2015 May 7;15:372. doi: 10.1186/s12885-015-1371-1 (PMC4427982; doi:10.1186/s12885-015-1371-1)
Supplement: Additional file 1: Figure S1. — Amino acid sequences of EpCAM variants. [file 12885_2015_1371_MOESM1_ESM.pptx]

## Slide 1
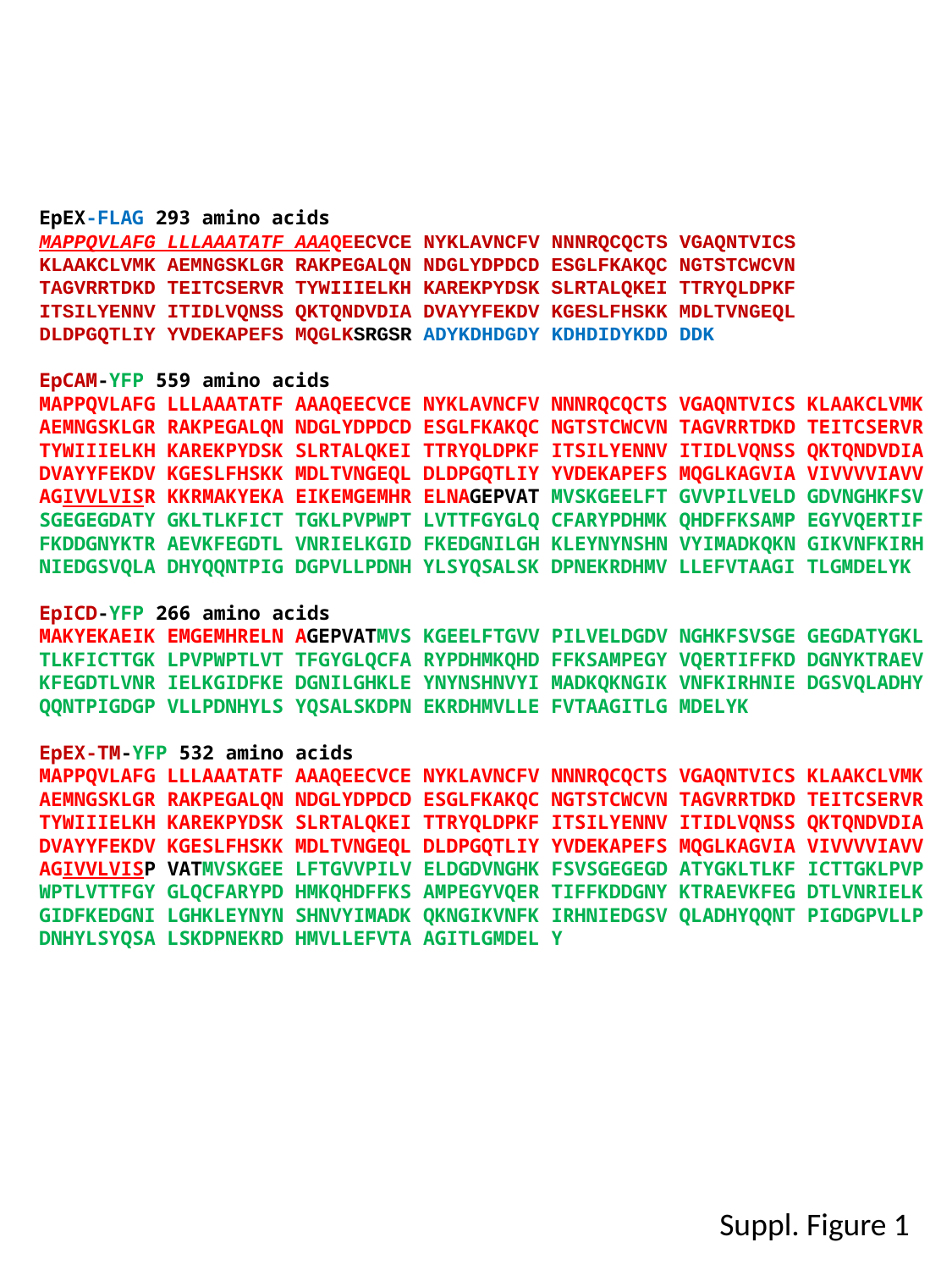

EpEX-FLAG 293 amino acids
MAPPQVLAFG LLLAAATATF AAAQEECVCE NYKLAVNCFV NNNRQCQCTS VGAQNTVICS
KLAAKCLVMK AEMNGSKLGR RAKPEGALQN NDGLYDPDCD ESGLFKAKQC NGTSTCWCVN
TAGVRRTDKD TEITCSERVR TYWIIIELKH KAREKPYDSK SLRTALQKEI TTRYQLDPKF
ITSILYENNV ITIDLVQNSS QKTQNDVDIA DVAYYFEKDV KGESLFHSKK MDLTVNGEQL
DLDPGQTLIY YVDEKAPEFS MQGLKSRGSR ADYKDHDGDY KDHDIDYKDD DDK
EpCAM-YFP 559 amino acids
MAPPQVLAFG LLLAAATATF AAAQEECVCE NYKLAVNCFV NNNRQCQCTS VGAQNTVICS KLAAKCLVMK
AEMNGSKLGR RAKPEGALQN NDGLYDPDCD ESGLFKAKQC NGTSTCWCVN TAGVRRTDKD TEITCSERVR
TYWIIIELKH KAREKPYDSK SLRTALQKEI TTRYQLDPKF ITSILYENNV ITIDLVQNSS QKTQNDVDIA
DVAYYFEKDV KGESLFHSKK MDLTVNGEQL DLDPGQTLIY YVDEKAPEFS MQGLKAGVIA VIVVVVIAVV
AGIVVLVISR KKRMAKYEKA EIKEMGEMHR ELNAGEPVAT MVSKGEELFT GVVPILVELD GDVNGHKFSV
SGEGEGDATY GKLTLKFICT TGKLPVPWPT LVTTFGYGLQ CFARYPDHMK QHDFFKSAMP EGYVQERTIF
FKDDGNYKTR AEVKFEGDTL VNRIELKGID FKEDGNILGH KLEYNYNSHN VYIMADKQKN GIKVNFKIRH
NIEDGSVQLA DHYQQNTPIG DGPVLLPDNH YLSYQSALSK DPNEKRDHMV LLEFVTAAGI TLGMDELYK
EpICD-YFP 266 amino acids
MAKYEKAEIK EMGEMHRELN AGEPVATMVS KGEELFTGVV PILVELDGDV NGHKFSVSGE GEGDATYGKL
TLKFICTTGK LPVPWPTLVT TFGYGLQCFA RYPDHMKQHD FFKSAMPEGY VQERTIFFKD DGNYKTRAEV
KFEGDTLVNR IELKGIDFKE DGNILGHKLE YNYNSHNVYI MADKQKNGIK VNFKIRHNIE DGSVQLADHY
QQNTPIGDGP VLLPDNHYLS YQSALSKDPN EKRDHMVLLE FVTAAGITLG MDELYK
EpEX-TM-YFP 532 amino acids
MAPPQVLAFG LLLAAATATF AAAQEECVCE NYKLAVNCFV NNNRQCQCTS VGAQNTVICS KLAAKCLVMK
AEMNGSKLGR RAKPEGALQN NDGLYDPDCD ESGLFKAKQC NGTSTCWCVN TAGVRRTDKD TEITCSERVR
TYWIIIELKH KAREKPYDSK SLRTALQKEI TTRYQLDPKF ITSILYENNV ITIDLVQNSS QKTQNDVDIA
DVAYYFEKDV KGESLFHSKK MDLTVNGEQL DLDPGQTLIY YVDEKAPEFS MQGLKAGVIA VIVVVVIAVV
AGIVVLVISP VATMVSKGEE LFTGVVPILV ELDGDVNGHK FSVSGEGEGD ATYGKLTLKF ICTTGKLPVP
WPTLVTTFGY GLQCFARYPD HMKQHDFFKS AMPEGYVQER TIFFKDDGNY KTRAEVKFEG DTLVNRIELK
GIDFKEDGNI LGHKLEYNYN SHNVYIMADK QKNGIKVNFK IRHNIEDGSV QLADHYQQNT PIGDGPVLLP
DNHYLSYQSA LSKDPNEKRD HMVLLEFVTA AGITLGMDEL Y
Suppl. Figure 1
