# Supplementary material for: Soluble EpCAM levels in ascites correlate with positive cytology and neutralize catumaxomab activity in vitro
Source: BMC Cancer. 2015 May 7;15:372. doi: 10.1186/s12885-015-1371-1 (PMC4427982; doi:10.1186/s12885-015-1371-1)
Supplement: Additional file 2: Material S1-S6. — Validation of the EpCAm ELISA. [file 12885_2015_1371_MOESM2_ESM.docx]

**Supplementary Material**

**Lower Limit of Quantification and Recovery Rates**

To determine the lower limit of quantification (LLOQ) in ascites we spiked recombinant EpCAM protein at concentrations of 75-5,000 pg in assay puffer (1% BSA) and in a pool of negative ascites (n=6). A seven point standard curve was used to compare results between different matrices. LLOQ was defined by a consistent signal with a precision of 20% 2-fold above the background signal of the zero samples in the respective biological matrix. Recovery rates were calculated with the corresponding optical densities of each standard in the ascites and BSA matrix.

**Inter-assay and Intra-assay Coefficient of Variation**

Positive ascites samples were used to test the intra- and inter-assay coefficient of variation (CV) to analyze the reproducibility of ELISA results. For the intra-assay variance we tested 3 low, 3 medium and 3 high ascites samples on 6 different locations on the same 96-well plate. For the inter-assay variance the same positive samples were tested three times on different 96-well plates, on different days by different operators.

**Stability of Ascites Samples**

Three low (<1,000 pg/mL), 3 medium (1,000 – 3,000 pg/mL) and 3 high (>3,000 pg/mL) positive ascites specimens were leaved unassisted for 2, 4, 8, 16 and 24 hours at room temperature to evaluate the short-term stability. Moreover, samples were exposed to repeated freeze-and-thaw cycles and then measured in the ELISA system. Degradation rates were calculated in comparison to initial samples. For the long-term stability, we measured these samples again after 9 months.

**Supplementary Material 1:** Recovery rates [% signal of O.D. 450] in ascites as biological matrix in comparison to standard in 1% BSA/PBS.

| **Standard** | **Concentration** | **BSA [%]** | **Ascites [%]** |
| --- | --- | --- | --- |
| STD 1 | 5,000 pg/mL | 100 | 110 |
| STD 2 | 2,500 pg/mL | 100 | 105 |
| STD 3 | 1,250 pg/mL | 100 | 106 |
| STD 4 | 625 pg/mL | 100 | 100 |
| STD 5 | 312 pg/mL | 100 | 86 |
| STD 6 | 156 pg/mL | 100 | 80 |
| STD 7 | 78 pg/mL | 100 | 85 |
| STD 8 | 0 pg/mL | 100 | 69 |
| **Mean** |  |  | **93%** |

**Supplementary Material 2:** Intra-assay coefficient of variation (CV) of 9 different ascites specimens on 6 different positions of the microtitre plate [pg/mL]. Mean ± Standard deviation (SD). Percentage (%) indicates difference of the sample between 6 positions.

|  | Position 1 | Position 2 | Position 3 | Position 4 | Position 5 | Position 6 | Mean | SD | % |
| --- | --- | --- | --- | --- | --- | --- | --- | --- | --- |
| Low 1 | 896 | 789 | 789 | 745 | 825 | 801 | 807.5 | 46.1 | 5.7 |
| Low 2 | 978 | 1,002 | 1,017 | 924 | 959 | 991 | 978.5 | 30.4 | 3.1 |
| Low 3 | 1,001 | 935 | 985 | 1,026 | 1,035 | 1,008 | 998.3 | 32.7 | 3.3 |
| Medium 1 | 1,866 | 1,931 | 1,832 | 1,902 | 1,852 | 1,950 | 1,888.8 | 42.4 | 2.2 |
| Medium 2 | 1,925 | 2,036 | 1,968 | 1,925 | 2,011 | 1,985 | 1,975.0 | 41.2 | 2.1 |
| Medium 3 | 2,136 | 2,203 | 2,283 | 2,111 | 2,247 | 2,196 | 2,196.0 | 59.2 | 2.7 |
| High 1 | 6,624 | 6,852 | 6,895 | 6,752 | 6,524 | 6,952 | 6,766.5 | 151.4 | 2.2 |
| High 2 | 5,484 | 5,236 | 5,147 | 5,589 | 5,214 | 5,410 | 5,346.7 | 158.8 | 3.0 |
| High 3 | 4,472 | 4,478 | 4,102 | 4,785 | 4,585 | 4,582 | 4,500.7 | 206.1 | 4.6 |
| **MEAN** |  |  |  |  |  |  |  |  | **3.2** |

**Supplementary Material 3:** Inter-assay coefficient of variation (CV) of 9 different ascites probes investigated by 3 operators on 3 different days [pg/mL]. Mean ± Standard deviation (SD). Percentage (%) indicates difference of the sample between 3 operators.

|  | Operator 1 | Operator 2 | Operator 3 | Mean | SD | % |
| --- | --- | --- | --- | --- | --- | --- |
| Low 1 | 896 | 935 | 1,004 | 945.0 | 44.7 | 4.7 |
| Low 2 | 978 | 958 | 1,059 | 998.3 | 43.7 | 4.4 |
| Low 3 | 1,001 | 1,029 | 905 | 978.3 | 53.1 | 5.4 |
| Medium 1 | 1,866 | 1,985 | 1,956 | 1,935.7 | 50.7 | 2.6 |
| Medium 2 | 1,925 | 1,901 | 2,241 | 2,022.3 | 154.9 | 7.7 |
| Medium 3 | 2,136 | 2,004 | 1,965 | 2,035.0 | 73.2 | 3.6 |
| High 1 | 6,624 | 6,321 | 6,898 | 6,614.3 | 235.7 | 3.6 |
| High 2 | 5,484 | 5,320 | 5,862 | 5,555.3 | 226.9 | 4.1 |
| High 3 | 4,472 | 4,822 | 4,925 | 4,739.7 | 193.9 | 4.1 |
| **CV (mean)** |  |  |  |  |  | **4.5** |

**Supplementary Material 4:** Influence of short-term stability of samples at room temperature [pg/mL]. Percentage (%) indicates difference of the sample between 0 and 24h timepoint.

|  | 0h | 4h | 8h | 16h | 24h | Degradation  after 24h | % |
| --- | --- | --- | --- | --- | --- | --- | --- |
| Low 1 | 896 | 921 | 841 | 871 | 785 | 111 | 12.4 |
| Low 2 | 978 | 954 | 932 | 901 | 849 | 129 | 13.2 |
| Low 3 | 1,001 | 1,019 | 984 | 965 | 921 | 80 | 8.0 |
| Medium 1 | 1,866 | 1,785 | 1,698 | 1,634 | 1,458 | 408 | 21.9 |
| Medium 2 | 1,925 | 2,001 | 2,098 | 1,958 | 1,842 | 83 | 4.3 |
| Medium 3 | 2,136 | 2,198 | 2,047 | 1,997 | 1,832 | 304 | 14.2 |
| High 1 | 6,624 | 6,541 | 6,258 | 6,487 | 6,024 | 600 | 9.1 |
| High 2 | 5,484 | 5,687 | 5,248 | 5,267 | 5,124 | 360 | 6.6 |
| High 3 | 4,472 | 4,256 | 4,025 | 4,036 | 3,941 | 531 | 11.9 |
| MEAN |  |  |  |  |  |  | **11.3** |

**Supplementary Material 5:** Influence on multiple freeze/thaw cycles on EpCAM protein stability in ascites as biological matrix. Concentrations [pg/mL].

|  | Before Cycles | After 3 Cycles | | Degradation | % |
| --- | --- | --- | --- | --- | --- |
| Low 1 | 896 | 836 | 60 | | 6.7 |
| Low 2 | 978 | 907 | 71 | | 7.3 |
| Low 3 | 1,001 | 952 | 49 | | 4.9 |
| Medium 1 | 1,866 | 1,702 | 164 | | 8.8 |
| Medium 2 | 1,925 | 1,854 | 71 | | 3.7 |
| Medium 3 | 2,136 | 1,985 | 151 | | 7.1 |
| High 1 | 6,624 | 6421 | 203 | | 3.1 |
| High 2 | 5,484 | 5,324 | 160 | | 2.9 |
| High 3 | 4,472 | 4,321 | 151 | | 3.4 |
| MEAN |  |  |  | | **5.3** |

**Supplementary Material 6:** Influence of long-term storage of samples (-20°C; pg/mL). Concentrations [pg/mL].

|  | Analysis | Analysis 6 months after | Degradation | % |
| --- | --- | --- | --- | --- |
|  |  |  |  |  |
| Low 1 | 985 | 896 | 89 | 9.0 |
| Low 2 | 1,136 | 978 | 158 | 13.9 |
| Low 3 | 1,598 | 1,001 | 597 | 37.4 |
| Medium 1 | 1,458 | 1,666 | -208 | -14.3 |
| Medium 2 | 1,845 | 1,925 | -80 | -4.3 |
| Medium 3 | 2,369 | 2,136 | 233 | 9.8 |
| High 1 | 6,584 | 6,624 | -40 | -0.6 |
| High 2 | 5,214 | 5,484 | -270 | -5.2 |
| High 3 | 4,783 | 4,472 | 311 | 6.5 |
| MEAN |  |  |  | **11.2** |
